# Indications and diagnostic value of bone marrow examination in HIV-positive individuals: A 3-year review at Tygerberg Hospital

**DOI:** 10.4102/sajid.v36i1.273

**Published:** 2021-08-23

**Authors:** Ibtisam Abdullah, Nadhiya Subramony, Ernest Musekwa, Erica-Mari Nell, Fatima Alzanad, Carissa Chetty, Ethan Gantana, Robert K. Lohlun, Wardah Cerfontein, Bridget Cochrane, Zivanai C. Chapanduka

**Affiliations:** 1 Department of Haematological Pathology, Stellenbosch University, Cape Town, South Africa; 2 National Health Laboratory Service, Cape Town, South Africa

**Keywords:** bone marrow examination, investigation of cytopenia, indications, diagnostic yield, diagnostic utility, HIV, lymphoma, leukaemia

## Abstract

**Background:**

Bone marrow examination is a useful diagnostic tool in human immunodeficiency virus (HIV)-positive patients presenting with cytopenias and fever. However, its role in the afebrile and asymptomatic patient presenting with an isolated cytopenia is not well established. This study was conducted to determine the indications for bone marrow examination and its diagnostic yield, in HIV-positive patients at Tygerberg Hospital.

**Methods:**

A retrospective, cross-sectional descriptive study was performed over a 3-year period from 01 September 2015 to 31 August 2018. The bone marrow examination reports for the HIV-positive patients who had a bone marrow examination during the study period were retrieved. Clinical and laboratory information was captured.

**Results:**

Altogether 374 bone marrow reports for HIV-positive patients were found. The indication of the bone marrow examination included investigation of unexplained cytopenias, suspected haematological malignancies, follow-up examination for patients with known haematological diseases, staging of haematological or non-haematological malignancies and investigation of suspected disseminated infection. The patients’ median age was 43 years and the interquartile range was 27–60 years. There was a slight female predominance with females 51% and males 49%. The diagnostic yield was 33.7%. Acute leukaemia and lymphoma were the most common diagnoses. Haematinic deficiency and pure red cell aplasia were found in the majority of cases with isolated anaemia. All cases with isolated thrombocytopenia were due to immune thrombocytopenia.

**Conclusion:**

Bone marrow examination is a useful investigation for HIV-positive patients with cytopenias, suspected haematological malignancy and lymphoma staging. However, its early use in patients with isolated anaemia and isolated thrombocytopenia is questionable.

## Introduction

According to the Joint United Nations Programme on human immunodeficiency virus (HIV) and acquired immunodeficiency syndrome (AIDS) (UNAIDS), there were an estimated 7.5 million people living with HIV (PLWH) in South Africa in 2018.^1^ South African guidelines published in 2017, recommend that all adult PLWH regardless of the CD4 count are eligible to receive anti-retroviral therapy (ART).^2^

Human immunodeficiency virus is known to cause several haematological changes.^3,4^ These include cytopenias, disseminated infections, immune thrombocytopenia (ITP), thrombotic thrombocytopenic purpura (TTP), thrombosis and a higher risk of haematological malignancies, particularly high-grade B-cell lymphoma.^3^ Cytopenia, the most common haematological complication of HIV, can be due to direct infection of the haematopoietic cells with cytokine dysregulation, ineffective haematopoiesis or because of peripheral destruction or loss of blood cells.^5^ Opportunistic infections such as mycobacterial and cryptococcal infections, with infiltration of the bone marrow, may occur. The microbial infiltration may be with or without granuloma formation and can also contribute to the cytopenias.

Other causes of cytopenia in PLWH include drugs, Parvovirus B19 infection, haematinic deficiency and bone marrow involvement with haematological and non-haematological malignancies.^6^ The most common cause of thrombocytopenia in PLWH is ITP, which is responsible for thrombocytopenia in up to 30% of the patients. Immune thrombocytopenia can be the first manifestation of HIV.^3^ Thrombotic thrombocytopenic purpura or TTP-like syndrome is prevalent in PLWH with a 15–40-fold increased risk compared with non-infected individuals.^7^ The risk of Non-Hodgkin lymphoma (NHL) is 60–200 folds higher in PLWH.^8^ The postulated mechanism of this increased incidence is chronic antigenic stimulation, cytokine dysregulation and co-infection by viruses such as Epstein–Barr virus (EBV) and human herpesvirus 8 (HHV8).^3^ In HIV-associated lymphomas, the evidence of viral coinfection can be demonstrated by immunohistochemical stains performed on bone marrow or other tissue. The immunohistochemical stains include EBV-encoded small RNA (EBER), latent membrane protein 1 (LMP 1) and HHV8 latency-associated nuclear antigen 1/2 (LANA1/2). Evidence of such co-infection can also aid diagnosis and classification of lymphoma.^8^

With such diverse possible causes of cytopenia, bone marrow examination is critically important in determining the aetiology of the cytopenias. Several studies have examined the role of bone marrow examination and its diagnostic utility in HIV-positive patients. The diagnostic utility of a test is determined by its technical reliability and cost-effectiveness. To assess the cost-effectiveness, one should take into consideration the diagnostic reliability and compare the cost between the different tests that provide similar clinical information. In addition, the clinical usefulness of the test is measured by whether the test changes the clinician’s diagnosis and/or management.^9^ One way of measuring clinical usefulness of bone marrow examination is by determining its diagnostic yield. The term diagnostic yield is variously defined. The most popular definition, which is used in this study, refers to the number and/or proportion of bone marrow examinations resulting in the establishment of a diagnosis and/or bone marrow involvement by any pathology.

Several studies aimed at determining the diagnostic yield of bone marrow examination found it to range from 27% to 47%.^3,8,9,10,11,12^ The diagnostic utility of bone marrow examination has been recently reviewed in the South African setting. Diagnostic utility in this context, is measured using the diagnostic yield and is defined as the number of diagnoses obtained from such a procedure.^10^ Bharuthram et al. estimated the diagnostic yield at 48% with 33% achieving a unique diagnosis. However, this study cannot be generalised to other units and hospitals as the study population only included patients who were admitted to the infectious disease (ID) unit.^10^ The diagnostic utility of this invasive procedure is still unclear, particularly when patients are afebrile and without clinical suspicion of haematological malignancies.

## Aim

The aim of the study was to determine the indications for bone marrow examination in the HIV-positive patients in our setting and to assess the diagnostic yield of bone marrow examination in these cases.

## Objectives

The objectives of this study were to:

- determine the demographic parameters for the study population
- evaluate the various full blood count parameters
- determine the various indication of bone marrow examination
- determine the final diagnosis for each bone marrow examination.

## Methods

The study was conducted at the National Health Laboratory Service (NHLS) Haematology laboratory at Tygerberg Hospital (TBH), a 1384-bed, multidisciplinary, tertiary care academic hospital in the Western Cape province of South Africa. Tygerberg Hospital is the main teaching hospital of the University of Stellenbosch Faculty of Medicine and Health Sciences.

This is a retrospective cross-sectional descriptive study of all confirmed HIV-positive patients whose bone marrow specimens were examined in the 3-year period from 01 September 2015 to 31 August 2018 regardless of age. Using the NHLS Laboratory Information System (LIS), all the bone marrow examination reports during the study period were captured by all investigators onto a Microsoft Excel^®^ database.

The following information was recorded:

- the age and sex of the patient
- the requesting doctor and/or clinical service
- the indication for the bone marrow examination
- the HIV status, CD4 count and HIV viral load
- the presenting FBC and pertinent peripheral blood smear findings
- the bone marrow aspirate and trephine (core) biopsy features
- the results of flow cytometry, immunohistochemistry, cytogenetics, polymerase chain reaction (PCR) and fluorescence in situ hybridisation (FISH)
- the final diagnosis.

The established database was verified and harmonised by one investigator to maintain consistency of terminology. A sub analysis was performed on the records of HIV-positive patients.

The StataCorp 2019 statistical analysis software was used for data analysis.^11^ The frequency and percentage were calculated for non-numerical parameters, whilst the median, mean and interquartile range were used for numerical parameters. The median was used for most haematological parameters because of the skewed distribution of the data. The mean was used for haemoglobin because of its symmetric distribution. The Mann–Whitney test was used to compare the haematological parameters between patients with CD4 count more than 200 cells/mm^3^ and patients with CD4 count less than 200 cells/mm^3^. A  *p*-value of less than 0.05 was considered statistically significant.

### Ethical considerations

The study was approved by the Ethics Committee of Stellenbosch University (project registration number HEA-8326 and ethics reference number N18/09/095) and was performed according to the Helsinki Declaration (2000) guidelines. A waiver of consent was obtained as the laboratory data were collected retrospectively. The laboratory data were collected by investigators to maintain confidentiality. Patients were identified by the allocated study number only. Patient confidentiality was maintained and patient identifying information was not included on the data capture sheet.

## Results

**FIGURE 1:**
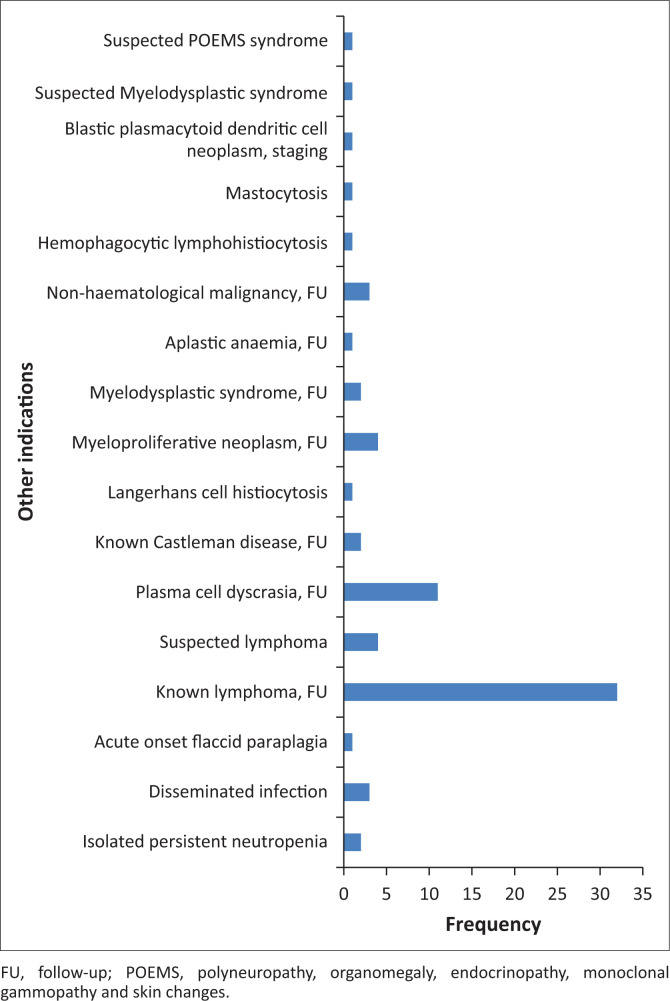
Detailed breakdown of the category ‘other indications’.

The total number of bone marrow samples examined during the 3-year study period was 2069, of which 374 (18%) were for confirmed HIV-positive patients. There were 192 females (51%) and 182 males (49%). The median age was 43 years and the interquartile range was 27–60 years. A CD4 count was available for 276 (72%) of the 374 HIV-positive patients and a viral load was available for 120 (32%). The indications for the bone marrow examination were widely variable and are summarised (Table 1). The large number of indications recorded as ‘Other indications’ are further characterised (Figure 1).

**TABLE 1:** Indications of bone marrow biopsies in the HIV-positive individuals.

Indication	Number of cases	%
Unexplained persistent cytopenias (bicytopenia or pancytopenia)	67	17.9
Unexplained isolated severe anaemia	16	4.3
Unexplained isolated thrombocytopenia	18	4.8
Known acute leukaemia, follow-up	44	11.8
Suspected new acute leukaemia	32	8.6
Suspected plasma cell neoplasms	24	6.4
Suspected lymphoproliferative neoplasm	16	4.3
Hodgkin lymphoma, staging	15	4
Low-grade non-Hodgkin lymphoma, staging	18	4.8
High-grade non-Hodgkin lymphoma, staging	39	10.4
Non-haematological malignancy, staging	14	3.7
Other indications including known lymphoma follow-up	71	19
**Total**	**374**	**-**

HIV, human immunodeficiency virus.

The diagnostic yield of bone marrow examination was 37.3% (*n* = 126). Follow-up cases of acute leukaemia (*n* = 44) and lymphoma (*n* = 7) were excluded when calculating the diagnostic yield. A list of diagnosis and their frequency is summarised (Table 2 and Table 3). Nine cases were suboptimal to make a definitive diagnosis because of inadequate material. Seven cases with cytopenia were diagnosed with lymphoma primarily on the bone marrow examination, in this study. Two of those were primary bone marrow Hodgkin lymphoma (HL). The others were three low-grade NHL, a plasmablastic lymphoma and a hepatosplenic T-cell lymphoma.

**TABLE 2:** The diagnostic yield of bone marrow examination.

Diagnosis	Number of cases	%
Acute leukaemia	35	27.8
Lymphoma	20	15.9
ITP	18	14.3
MDS or MPN	19	15.1
PRCA	4	3.1
Plasma cell dyscrasia	19	15.1†
CLPD	7	5.5
Tuberculosis	3	2.4
Involvement by non-haematological malignancy	1	0.8
**Total**	**126**	-

ITP, immune thrombocytopenic purpura; MDS, myelodysplastic syndrome; MPN, myeloproliferative neoplasms; PRCA, pure red cell aplasia; CLPD, chronic lymphoproliferative disorders.

†, Plasma cell dyscrasia cases include a single case of plasma cell leukaemia.

**TABLE 3:** Diagnostic yield of bone marrow examination by indication.

Indication	Final diagnosis (number of cases)
Cytopenias	Lymphoma (7) Acute leukaemia (5) Aplastic anaemia (5) Haematinic deficiency (3) Granuloma (3) MDS (2) MM (2) HCL (1) HIV-related ineffective haematopoiesis, sepsis, peripheral causes of cytopenia (36)
Unexplained isolated anaemia	PRCA (4) Haematinic deficiency (5) Multifactorial (4) Bone marrow involvement by haematological malignancies (2) MDS (1)
Unexplained isolated thrombocytopenia	ITP (18)
Lymphoma, staging or follow-up	Involved (15) Not involved (52)
Suspected new acute leukaemia	Acute leukaemia (31) MDS (1)
Suspected plasma cell neoplasms	MM (16) Plasma cell leukaemia (1) TB granuloma (1) Normal bone marrow findings (5)

MDS, myelodysplastic syndrome; MM, multiple myeloma; HIV, human immunodeficiency virus; PRCA, pure red cell aplasia; ITP, immune thrombocytopenic purpura; TB, tuberculosis.

Acute leukaemia contributed the most to the diagnostic yield, 27.8% (Table 1). A case of plasma cell leukaemia was included in the plasma cell dyscrasia category (Table 1), which makes the total number of acute leukaemia cases, 36. Acute myeloid leukaemia represented the majority of acute leukaemias 58.3% (*n* = 21/36) followed by lymphoblastic leukaemia, 38.9% (*n* = 14/36) and plasma cell leukaemia, 0.03%. The majority of acute lymphoblastic leukaemias were of B-cell lineage (*n* = 12).

Various haematological parameters of HIV-positive patients who underwent bone marrow examination were evaluated and summarised (Table 4).

**TABLE 4:** Haematological parameters of the study population on presentation.

Haematological parameters	Mean/Median	SD/IQR
Haemoglobin (g/dL)	9.7	2.8
White cell count (× 10^9^/L)	6.1	3.71–10.04
Platelet count (× 10^9^/L)	197	80.5–334.5
Absolute neutrophil count (× 10^9^/L)	3.1	1.6–5.4
Lymphocytes count (× 10^9^/L)	1.5	0.85–2.4
Monocytes count (× 10^9^/L)	0.4	0.19–0.7
Eosinophils count (× 10^9^/L)	0.1	0.04–0.19
CD4 counts (cells/mm^3^ )	184.0	97–364
Viral load (copies/mL)	23.0	20–5882

SD, standard deviation; IQR, interquartile range.

The patient population was divided into two groups based on the CD4 count. Group-1 had CD4 < 200 cells/mm^3^ and group-2 with CD4 > 200 cells/mm^3^. Median absolute neutrophil count in the category of patients with CD4 < 200 cells/mm^3^ was higher than that of CD4 > 200 cells/mm^3^. The difference was statistically significant (*p* = 0.04) using Mann–Whitney U test. There was no statistically significant difference in the other haematological parameters between the two categories as shown (Table 5).

**TABLE 5:** Comparison of haematological parameters in patients with different CD4 counts (Mann–Whitney U test).

Haematological parameters	CD4 count < 200 cells/mm^3^		CD4 count > 200 cells/mm^3^		*p*
	Mean/Median	SD/IQR	Mean/Median	SD/IQR	
Haemoglobin g/dL	9.20	2.9	9.3	2.7	0.84
White cell count (× 10^9^/L)	6.10	3.65–9.87	5.53	3.72–10	0.52
Platelet count (× 10^9^/L)	186	71–321	203	91–351	0.39
Neutrophil count (× 10^9^/L)	3.26	1.62–6.0	2.68	1.4–4.4	0.04
Lymphocyte count (× 10^9^/L)	1.44	0.8–2.2	1.67	0.94–2.7	0.18
Monocyte count (× 10^9^/L)	0.40	0.2–0.75	0.36	0.19–0.68	0.62

SD, standard deviation; IQR, interquartile range.

Granuloma formation was seen in 28 (7.5%) of the 374 bone marrows examined. The Ziehl Nielsen (ZN) and/or Periodic Acid Schiff (PAS) stain was positive in 11 (39.2%) of 28 indicating bone marrow infection with Mycobacterium tuberculosis or atypical mycobacteria. Nine of these cases had other diagnoses in conjunction with tuberculosis, whilst in three cases mycobacterial infection was the only diagnosis made on the bone marrow. Of the patients who had granulomata, only two patients had a CD4 count available.

## Discussion

In this study, the study population included all HIV-positive patients who had bone marrow examination during the study period, thus it represents an unselected population. Other studies focussed on the diagnostic yield of bone marrow examination in HIV-positive patients with fever and/or cytopenia or HIV-positive patients from a selected ward with defined admission criteria.^4,10^ Studying the indications and the diagnostic yield of bone marrow examination in unselected HIV-positive patients provides an additional advantage to previous studies by examining the diagnostic utility of bone marrow examination in afebrile HIV-positive patients. This difference in the study population may explain the major differences in our results compared with other South African studies.

The indications for bone marrow examination were widely variable in this study, unlike other South African studies.^4,10^ The most common indications were bicytopenia or pancytopenia. The second most common indication was assessment of known cases of acute leukaemia. Staging of new cases with high-grade lymphomas was the third commonest indication. Performing bone marrow examination to investigate patients with fever of unknown origin (FUO) was underrepresented in our study. As a result of the wide range of indications of bone marrow examination in this study and the limited number of cases with FUO, the findings of this study should be compared with caution with other studies, which focused on the diagnostic utility of bone marrow examination in patients with fever and/or cytopenia.

The term diagnostic utility includes the diagnostic yield and cost-effectiveness of a test. However, some investigators use the term diagnostic utility to refer to diagnostic yield. In this study, we prefer the term diagnostic yield to describe the number of diagnoses established from the bone marrow examination. There are few reports that examined the diagnostic yield of bone marrow examination in unselected patients with HIV. To our knowledge, this is one of the largest studies that were performed to determine the diagnostic yield of bone marrow examination in an unselected HIV-positive population.

The diagnostic yield of bone marrow examination in HIV-positive patients was 37.3%, which is in agreement with previous studies.^12,13^ A few local studies had a higher diagnostic yield (approximately 48%) for HIV-positive patients in ID unit and patients with fever and/or cytopenia.^4,10^ The difference in diagnostic yield in this study compared with other South African studies is predominantly because of different study design. In this study, the samples originated from a wide variety of clinics and wards and not only from an ID setting or solely for the investigation of fever.

In the other South African studies, the most common diagnoses were disseminated mycobacterial infections and ITP.^4,10,12^ In this study, the most common diagnoses were acute leukaemia followed by lymphoma. The incidence of acute leukaemia in PLWH is uncertain because of limited epidemiological data. A meta-analysis study of the incidence of cancer in PLWH found an increased leukaemia incidence in this population. However, the exact aetiology of this increased incidence or a link between HIV and specific leukaemia type remains to be defined.^14^ The high incidence of acute leukaemia in our study could be because of higher incidence of leukaemia in PLWH and not as a result of HIV infection per se. It probably parallels the high incidence of leukaemia diagnosis in general population in our institution. A follow-up study comparing the diagnostic yield of bone marrow examination in HIV-positive and non-infected patients would be valuable to explore significance of these findings at our institution. The low frequency of disseminated mycobacterial infections in this study could be explained by several reasons. Firstly, bone marrow examination was rarely performed to investigate pyrexia of unknown origin (PUO) or suspected mycobacterial infections, which represented a high percentage in the other South African studies 40.8%, 22% and 32% in Karstaedt et al. 2001, Van Schalkwyk et al. 2011 and Bharuthram et al.^10^, respectively. Secondly, in this study, the diagnosis of disseminated mycobacterial infection rested solely on histological examination of the trephine biopsy. Diagnosis by TB culture was not included in this study as it was in the other studies. Granulomata were described in 28 bone marrow samples. The ZN and/or PAS stain was employed to identify both atypical mycobacterium and fungal infections in cases of suspected bone marrow granulomas. The ZN and/or PAS stains were positive in 11 (39.2%) of 28 confirming mycobacterial infection in these cases. Reduced CD4 counts are associated with poor granuloma formation, especially if the CD4 count is < 50 cells/mm^3. 15^ The poor granuloma formation could be explained by paucibacillary specimens and/or the inability of the immune system to mount a granulomatous reaction in severely immunocompromised patients.^16^ Meaningful statistical correlation between CD4 count and granuloma formation was not possible in this study because of the limited availability of CD4 count data.

Bone marrow examination performed to investigate two or more cytopenias showed a unique diagnosis in 37.3% of the cases. Bone marrow involvement by lymphoma was the most common unique diagnosis in HIV-positive patients presenting with two or more cytopenias. This is followed by acute leukaemia and aplastic anaemia. More than half (58.2%) of the cytopenias were because of HIV-related ineffective haematopoiesis, sepsis, peripheral destruction/sequestration or haematinic deficiency. Therefore, bone marrow examination has high diagnostic utility in HIV patients presenting with unexplained cytopenias. The diagnostic utility is higher once sepsis, haematinic deficiencies and myelotoxic drugs have been excluded as cause for the cytopenia.

Bone marrow examination performed to investigate severe, unexplained, isolated anaemia showed a unique diagnosis in 7 (43.8%) of 16 cases. Haematinic deficiency was the commonest cause of anaemia in this population, seen in 5 (31.3%) of 16 cases followed by pure red cell aplasia (PRCA).

A positive PCR analysis for Parvovirus B19 was found in 4 (25%) of 16. Overall, haematinic deficiency and PRCA constituted more than half of the causes of isolated anaemia in this study.

Haematinic studies and PCR analysis for Parvovirus B19 are more convenient, safer and more cost-effective than a bone marrow examination. Therefore, it is recommended to perform haematinic studies and PCR for Parvovirus B19 on peripheral blood sample before requesting bone marrow biopsy to investigate severe unexplained isolated anaemia in HIV-positive patients.

The prevalence of thrombocytopenia is 30% – 40% amongst PLWH.^17^ The diagnosis of ITP in HIV-positive patients with fever and/or cytopenia was made in 21% of the bone marrow cases in a previous local study, compared with 14.3% in this study.^4^ However, in both studies, all bone marrow examination to investigate unexplained thrombocytopenia showed an increase of megakaryocytes consistent with the diagnosis of ITP. There is, therefore, little or no value in performing an invasive bone marrow examination in HIV-positive adult patients with isolated thrombocytopenia.

The prevalence of bone marrow involvement by all lymphomas in this study was 20.8%, which is higher than other local studies, mostly because of differences in study design.^4,10,12^ It is well recognised that there is a significant rate of lymphomatous bone marrow involvement in HIV-associated lymphomas.^18,19^ A previous local study showed that bone marrow involvement by HL was 45.5% in HIV-positive individuals versus 19.5% in HIV-negative individuals.^19^ Identification of patients with bone marrow involvement has prognostic and therapeutic implications.^20^ Positron emission tomography–computed tomography (PET–CT) scan appears to identify bone marrow involvement by high-grade lymphoma with high sensitivity given its high negative predictive value. Although PET–CT scan could be considered as an alternative to bone marrow examination, further studies are needed to determine its role in HIV-positive individuals.^21^ Primary bone marrow HL, which has an aggressive clinical course, generally presents with cytopenias and B symptoms.^3^ Seven cases with cytopenia were diagnosed with lymphoma primarily on the bone marrow examination, in this study. Two of those were primary bone marrow HL.

The risk of plasma cell disorders is higher in HIV-positive individuals.^22,23,24^ An international study that linked cancer registries in the United States of America, Italy and Australia revealed a relative risk of multiple myeloma in HIV/AIDS ranging from 2 to 5.^25^ Plasma cell dyscrasia constituted 15% of the diagnostic yield in our study compared with 0.39% in another South African study.^12^ This difference could be because of the increasing use of ART with an increased median age of PLWH and increased risk of development of haematological malignancies.

The strength of this study is that it included all HIV-positive patients who had bone marrow biopsy performed at TBH during the study period, with no selection bias. Other local studies focussed on bone marrow biopsies performed because of specific pathology such as cytopenia and FUO.^4,10^ A single local study that focussed on usefulness of bone marrow biopsy regardless of the indication was performed before the introduction of anti-retroviral treatment for HIV.^12^

Most haematological parameters were not significantly different in HIV-positive individuals with CD4 less than 200 cells/mm^3^ and those more than 200 cells/mm^3^. Interestingly, neutrophils count was significantly higher in patients with CD4 count less than 200 cells/mm^3^. It was observed that 6 of these patients who had CD4 less than 200 cells/mm^3^ had absolute neutrophil count more than 50 000, which may have resulted in skewing of data. Five of these patients had a myeloproliferative neoplasm and the sixth patient had reactive neutrophilia. Changes in haematological parameters in HIV-positive patients in relation to CD4 count were variable in previous studies.

Studies comparing the haematological parameters in HIV-positive patients with CD4 counts in the ranges < 200, 200–400 and > 500 cells/mm^3^ found significant difference in total leucocyte count between these three groups.^26,27^ A study by Parinitha et al. found significant difference in absolute lymphocyte count between patients with different CD4 count whilst Rahman et al. showed that there was no significant difference in absolute lymphocyte count between patients with the various CD4 count values. Both studies did not compare monocyte count.^26,27^

Limitations of this study include its retrospective nature, collecting data available on the bone marrow reports only might cause information bias because of lack of clinical data and other laboratory data such as the results of blood or sputum TB cultures.

## Conclusion

This study highlights the important role of bone marrow examination in unselected HIV-positive individuals presenting with two or more cytopenias, lymphoma, suspected leukaemia or plasma cell disorders. However, the usefulness of bone marrow examination is questionable in HIV-positive individuals presenting with isolated anaemia or thrombocytopenia. In these patients, safer and more cost-effective tests such as haematinic assays and Parvovirus B19 PCR should be performed first.
